# Bearing Properties of CFRP Composite Laminates Containing Spread-Tow Thin-Plies

**DOI:** 10.3390/polym14102076

**Published:** 2022-05-19

**Authors:** Hassan Alshahrani, Tamer A. Sebaey

**Affiliations:** 1 Department of Mechanical Engineering, College of Engineering, Najran University, Najran 11001, Saudi Arabia; 2 Engineering Management Department, College of Engineering, Prince Sultan University, Riyadh 12435, Saudi Arabia; 3 Mechanical Design and Production Department, Faculty of Engineering, Zagazig University, Zagazig 44519, Egypt

**Keywords:** CFRP composite laminates, bearing, bolted joints, damage mechanisms, spread-tow thin-plies

## Abstract

With the development of spread-tow, thin-ply technology, ultra-thin composite laminates could be produced. Composite bolted joints are commonly used on aircraft’s load-bearing structures and are considered the main cause of stress concentration. The aim of this research is to investigate the bolted joint behavior of composite laminates that combine thin-plies and conventional thick-plies in a predetermined stacking sequence. The impact of thin-ply placement within the stack on bearing strength, including the onset of damages, is examined. The work involves mechanical tests and fractographic activities to understand the damage mechanisms of the plies and their interactions, and its reflections on the bearing load capacity of the joint for double-lap bolted joints. The results showed an improvement in the bearing strength of up to 19% by inserting the thin-plies inside the laminate. The visual examination of the specimens showed a bearing damage mode for all the tested specimens. The computed tomography scans showed damage mechanisms that mostly occurred with the normal plies, rather than breaking the thin-plies. For the specimens of traditional plies, delaminations were noticed at most of the interfaces. For the one with a block of thin-plies in the middle, all the delaminations were forced to the surface layers with an extra large size. Forspecimens with distributed thin-plies, a higher number of smaller delaminations was recognized.

## 1. Introduction

Due to their higher specific modulus and strength, fatigue resistance, and light weight compared to traditional materials, carbon-fiber-reinforced polymer (CFRP) composites are in a constant state of growth for several applications. In addition, the use of CFRP composites as the main load-bearing structures gradually increased in the aerospace industry. Conventional, well-known composites are made of several layers of fiber-reinforced plastics of a nominal ply thickness greater than 0.1 mm [1]. Reducing the ply thickness below this limit is usually referred to as thin-plies. In 2007, Tsai and Kawabe [2] published a patent that addressed a new tow spreading technology that enables the production of plies of thicknesses ranging down to 0.02 mm. They showed some of the potential improvements in the structural behavior using thin-ply laminates. The growing interest in thin-plies is justified by the possibility of improving the individual ply’s in situ strength [3,4,5].

Yokozeki et al. [6] studied the damage characteristics of standard and thin-ply laminates under transverse loading. Owing to the positive effects of the thin-ply composite laminates, such as a lower ply thickness and reduced delamination and matrix cracking before ultimate failure, the damage and fatigue resistance improved as compared to the conventional composites. Arteiro at al. [7] summarized these advantages as a higher number of plies per laminate thickness, which ultimately reduced the final composites’ thickness, makign them lighter structures as compared to the traditional plies’ composites.

The primary and secondary load-bearing aircraft structures have several composite bolted joints [8]. However, these joints are commonly the weakest area in a composite structure and this kind of material lacks the ability to redistribute local high stresses. This is why joint design is especially important in fiber-reinforced composite structures [9,10]. To ensure the practicability of fiber-reinforced composite structures in aircraft structures, it is essential to understand both the damge initiation and propagation and the failure sequence of composite bolted joints [11]. Bearing, shear-out, and net-tension failures are the three key failure modes in mechanically fastened joints [12]. Other types of failure modes can arise by combining these failure types, such as tension with shear-out and wedge-type splitting [13]. Due to the disastrous final failure, net tension and shear out failure types are not preferred.

The behavior of composite bolted joints is governed by a number of variables. The joint design (single- and double-lab), the specimen’s geometrical parameters, the clamping torque, the hole-cutting parameters, the washer size, and the bolt/hole clearance are all important aspects to consider. The influence of geometric aspects on the failure modes and loads of carbon/epoxy double-lap composite bolted structures joints was numerically and experimentally examined by Zhou et al. [14]. It has been reported that increasing the width of composite samples with bolted joints will dramatically increase the composite specimen’s failure load. The increase in the edge distance (E) value had a noticeable impact on the improvement in the failure load when W/D = 4 and E/D is less than 3. When the E/D ratio is higher than 3, increasing E does not affect the failure load. In the single-lap composite joints of carbon-fiber reinforced by BMI resin, Li et al. [15] reported that increasing the W/D ratio reduced the effect of subordinate bending and switched the fracture type from bearing to net-section failure. The influence of clamping torque on the bearing behavior including double-lap bolted joints bearing strength was investigated by Wang et al. [16]. It was concluded that a high preliminary clamping pressure improved bearing properties by preventing the generation of shear cracks in the laminate. Bolt torque reduces joint stiffness damage and greatly improves bearing strength [17]. The influence of washer size on the bearing load capability of glass-fiber-reinforced polymer (GFRP) composites was investigated by Khashaba et al. [18]. As a consequence of increasing the compressed area under the washer, the slope in the load-displacement diagram of bolted joints (stiffness) rises with reductions in the washer size under the same gripping torque. Yoon et al. [19] explored the single-lap bolted joint design experimentally after optimization and a numerical prediction of its bearing behavior. By increasing the tightening torque, the desired bearing failure mechanism and bearing strength were detected. However, a large increase in tightening torque could cause initial cracking, resulting in a reduction in bearing strength [20]. For glass/epoxy laminates, Khashaba et al. [21] explored the role of bolt/hole clearance on bearing strength and failure modes in glass/epoxy laminates. As compared to samples without clearance, raising the pin–hole clearance up to 300 μm caused a drop in bearing strength. They explained this finding by the reduction in the pin hole contact area, which caused the harm region to be more concentrated, leading to increased radial and bearing stresses. Among the different parameters, Choudhury and Debnath [20] showed that the W/D ratio is the most important parameter, following this the bolt-fastening torque, and, finally, the E/D ratio.

Reducing the thickness of single ply is one method of delaying damage creation that has been described in the literature. The obtained developments in strength are denoted as the in situ effects. The effects of thin-ply composites have been experimentally observed and revealed for a variety of loading scenarios [6,22,23,24,25]. In some applications, brittle fracture governed the whole story and yielded undesirable behavior. In others, the behavior of the composites was improved. The improvement was due to the stress relaxation and the energy release rate at the tip of the crack, as was shown by the meso-scale finite-element modeling [26]. In addition, thin-plies can resist the propagation of large cracks, which contributes to this improvement [7].

Research on the damage mechanisms of bolted joints’ connections in composite structures containing thin-plies are limited in the literature. Cameron et al. [27] studied the effect of using thin-plies with and/or instead of the traditional plies. The results showed an increase in the bearing stiffness, strength at onset of damage, and ultimate bearing stress by increasing the amount of thin plies within the laminate. Moving from from a full traditional ply laminate to a full thin-ply laminate resulted in a 47% increase in the onset bearing strength. Single-lap joints loaded in tension with a combination of thick and thin plies of various materials showed some interesting increases in strength [28]. During quasi-static loading, the bearing behavior of thin-pies was investigated by Cao et al. [29] using a double lab joint. Hybrid joint damage was caused by a combination of hole-bearing deflection and bolt tensile cracking. Thin-ply laminate bearing failure was caused by a complex blend of four damage modes: wedge matrix, fiber kinking, cracking, fiber/matrix fracturing, and crushing. Thin-ply laminates had their delamination damage and tensile mechanisms suppressed, leading to an increased bearing failure strength and further damage accumulation. A similar study was carried out by Cao et al. [30], but for a single lab joint. The main damage modes of thin-ply samples were found to be close to those of conventional-thickness composites, with a minimal delamination area upon the usage of thin-plies. This phenomenon can be justified by the increased number of interfaces in thin plies which results in higher number of small delaminations [31].

In the current study, the objective is to experimentally investigate the bolted joint behavior of a composite that contains thin-plies with traditional thick-plies in a designed stacking sequence. The work includes mechanical tests as well as delamination observations through a computed tomography scan to understand the damage mechanisms of the plies and their interactions and effects on the bearing load capacity of the joint.

## 2. Materials and Manufacturing

In the current study, two grades of woven carbon fibers fabrics, supplied by Oxeon, were used. The identifications of the two fabrics are TeXtreme 80 PW UTS50S of 80 gsm and TeXtreme 320 PW UTS50S of 320 gsm. The first fabric grade was 80 gsm, with 40 gsm in each direction. This resulted in a layer of 21 μm, which can be classified as thin-ply. One TeXtreme fabric layer is equivalent to 4 layers of unidirectional fabric. The nominal ply thickness of the traditional plies was 330 μm whereas, for the thin-plies, this was 85 μm. Autoclave manufacturing process was used to manufacture the plates with MTFA500 (DF044) epoxy resin (delivered by SHD composites).

**Figure 1:**
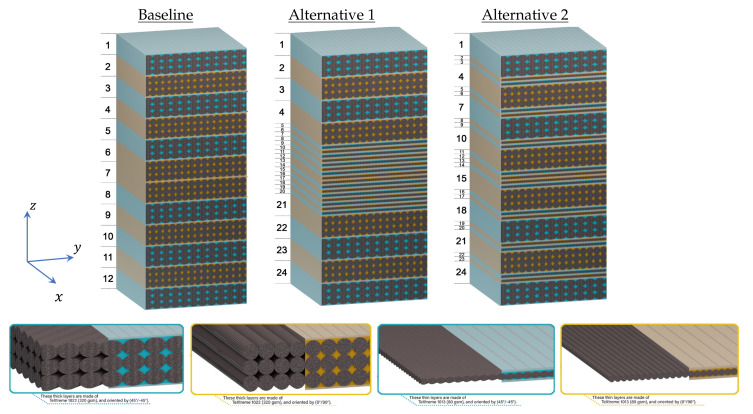
Schematic of the cross-section properties of the three stacking sequences.

Considering the nature of damage to bolted joint problems and to avoid brittle failure, hybrid specimens were manufactured from both traditional and thin-plies, and the bearing response was compared with specimens made of all traditional plies. Three stacking sequences were considered. The first stacking sequence was defined as the baseline or the reference laminate, which consists of 12 layers of TeXtreme 320 PW. To ensure the quasi-isotropic characteristic of the laminate, during the design stage all samples had the same amount of fiber in 0${}^{\circ }$, 90${}^{\circ }$, 45${}^{\circ }$, and −45${}^{\circ }$ directions, Figure 1. The total laminate thickness was 3.98${}^{\pm 0.038}$ mm.

The two other stacking sequences were designed with both the thin and thick plies. The first one (alternative 1) had traditional layers at the specimen surfaces and a block of thin-plies at the mid-plane. The design criteria of this laminate are force the damage at the specimen’s surfaces and maintain a cluster of layers with minimal damage at the specimen’s mid-plane. This cluster can delay the final breakage of the specimen and, consequently, improve maximum strength and reduce the possibility of catastrophic failure. To be able to compare this with the baseline, the amount of fiber in all direction was the same as in the baseline. The total thickness of this laminate was 4.10${}^{\pm 0.027}$ mm. The second alternative was designed with the in situ strength phenomenon. It is well-known that the crack initiation/propagation is highly affected by the neighboring plies’ strength (i.e., the higher the strength and stiffness of the neighboring plies, the higher the damage resistance of the ply under consideration) [26]. For the alternative 2 laminates, the design criteria are to have each traditional ply surrounded by two thin-plies, to improve its in situ strength. The total thickness of the second laminate configuration was 4.11${}^{\pm 0.033}$ mm. The stacking sequences of the three laminates are (the superscript *T* refers to thin-ply):

- Baseline ${\left[\left(\pm 45\right)/\left(0/90\right)\right]}_{3S}$
- Alternative 1 ${\left[{\left[\left(\pm 45\right)/\left(0/90\right)\right]}_{2}/{\left[{\left(\pm 45\right)}^{T}/{\left(0/90\right)}^{T}\right]}_{4}\right]}_{S}$
- Alternative 2 ${\left[\left(\pm 45\right)/{\left(0/90\right)}^{T}/{\left(\pm 45\right)}^{T}/\left(0/90\right)/{\left(\pm 45\right)}^{T}/{\left(0/90\right)}^{T}\right]}_{2S}$

The thickness of the two alternatives was greater than that of the baseline. This increase in the thickness is due to the higher number of interfaces in the laminates that contain thin-plies. It is worth remarking that this increase in the thickness cannot be avoided. However, it is believed that this increase in the thickness does not significantly affect the bearing strength because it is relatively small (2.5%) and the interface layer that causes this increase is made of a pure matrix.

With those two designs alternatives, we could benefit from the improved strength of the thin-plies and avoid the brittle nature associated with laminates made entirely thin-plies. The three plates were manufactured with 700 × 700 mm. The margins, of 40 mm, were cut using computer-controlled milling cutter. The fiber volume fraction was measured by the ignition test standard, ASTM D2584-11. The values of the fiber volume fraction were 50.41${}^{\pm 0.52}$%, 49.09${}^{\pm 1.03}$% and 50.01${}^{\pm 0.28}$% for the baseline, alternative 1 and alternative 2, respectively.

## 3. Sampling and Test Methods

### 3.1. Double-Lap Bolted Joints

**Figure 2:**
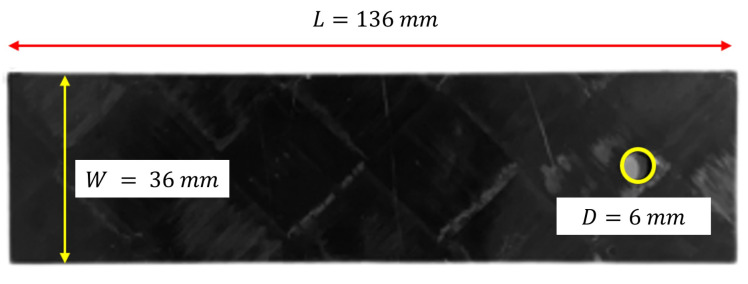
Sample’s dimensions for double-lap bolted joint.

The ASTM D5961 test standard was used to carry out double-lap bolted-joint tests. The bolt diameter (*D*) was 6 mm. The edge-distance-to-diameter ratio E/D was 3 and the width-to-diameter ratio W/D was 6 for all specimens, as shown in Figure 2. To obtain the final dimensions of the specimens, a cutting process was used with a CNC milling machine of 3-mm diameter end-mill and predefined cutting parameters, as per the guidelines in [32].

**Figure 3:**
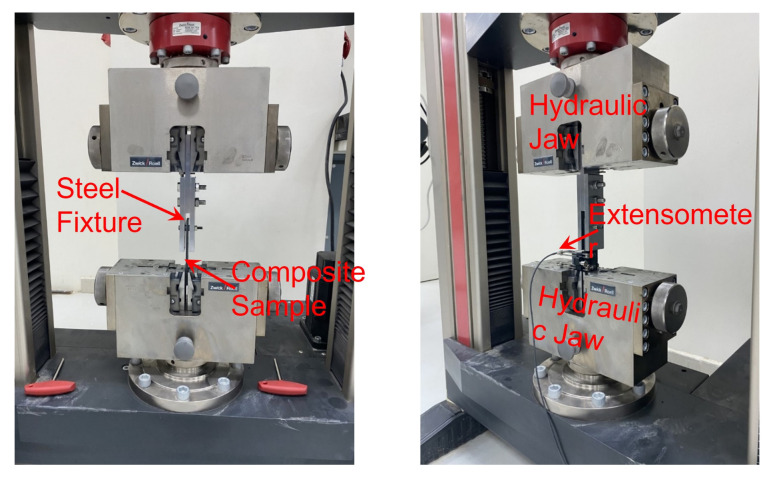
Experimental setup and test fixture for double lap bolted joints.

Stainless steel test fixture was manufactured according to the geometries indicated in the test standard Method A. Figure 3 shows the experimental setup and test fixture for double-lap bolted joints. Each specimen was attached to the upper cross-head via a pin running through the sample’s hole. The sample was then centered and clamped in the bottom cross-head. To ensure the setup remained unloaded before testing began, the testing software performed an automatic de-loading of the cross-head while tightening the specimen’s clamps. Once clamped, the extensometer was attached to directly measure the strain. The displacement was slowly increased at 2 mm/min to discard non-linearity and erratic values at the test’s start.

During the test, the machine reads the load from the load cell and the extensometer reads and process the deformations to obtain the strain. The bearing strain at any datapoint is calculated as:

(1) $$\epsilon_{i}^{br}=\frac{\delta_{i}}{K\cdot D}$$

where, $\delta_{i}$ is the extensometer displacement at the ith datapoint and *K* is a constant equals 1 for double shear and 2 for single shear. The bearing stress for the same datapoint is calculated as follows:

(2) $$\sigma_{i}^{br}=\frac{F_{i}}{k\cdot D\cdot t}$$

where, $F_{i}$ is the force reading at the *i*th datapoint and *t* is the thickness of the sample. In the literature, a common practice is to have a drop in the load profile at the initiation of the bearing failure. This datapoint is usually characterized by the bearing strength. Whereas, at the maximum load capacity of the specimen, the stress measured is usually addressed as the ultimate strength [13]. The point corresponding to the initiation of the bearing failure can also be determined from the offset strength at 10% of the bearing strain, in case it is not clearly defined by a drop in the load-displacement curve, considering the mounting and data of the extensometer.

### 3.2. Computed Tomography Scan

One sample of each plate was cut into two identical halves after being tested, using the water-jet cutting. Half of the sample was vertically mounted inside the Zeiss Xradia 510 Versa CT scanner. The X-ray voltage and current were 80 kV and 87 μA, respectively. The pixel size was 0.142 mm and each individual image was 2646 × 2646 pixels. The resulting field of view was a cube with a 19 mm side length. In total, 2001 projections were collected from each scan, with an exposure time of 5 s, and a sample rotation of 360${}^{\circ }$, resulting in a total scan time of 1.2 h. The result of this step was a series of images (hundreds of images) through the specimen cross-section. The images were then reconstructed in 3D. The images were compared at different locations and orientations of the three configurations to assess the delaminations.

## 4. Results and Discussions

**Figure 4:**
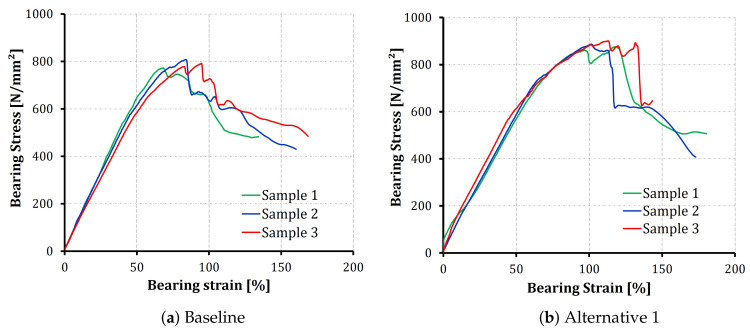
Bearing stress-bearing strain curves of the baseline and the A1 configurations.

**Figure 5:**
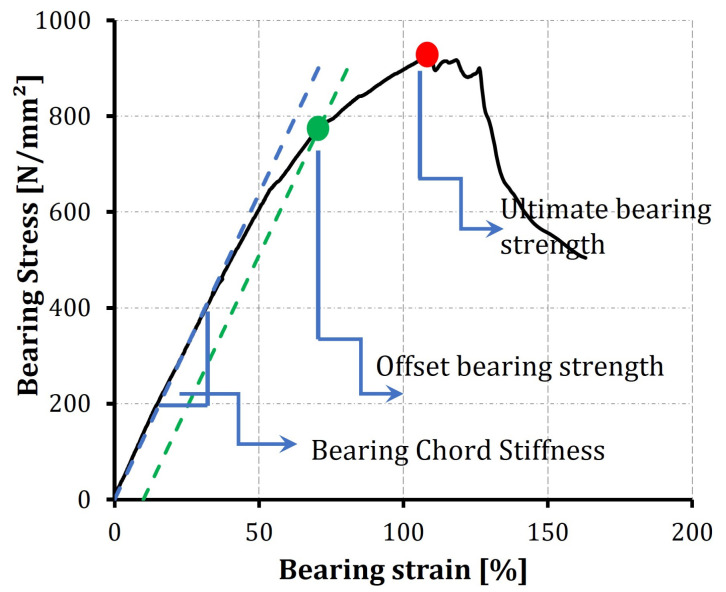
Bearing stress-bearing strain curves of the Alternative 2 Sample 1 with the most common notation.

At least three samples were tested with each configuration. Figure 4 shows the bearing stress vs. bearing strain diagrams for three specimens of the baseline and A1 configurations. For the plots shown in Figure 4, the bolted joint tests show excellent repeatability, especially before the failure is initiated. All the samples of all the configurations followed the same bearing stress-bearing strain profile. Figure 5 shows details of the bearing stress-bearing strain curve for one of the alternative 2 samples, for illustration of the measurements.

The first stage in the bearing stress-strain profile shows a linear response, which reflects a pure deformation with no significant damage initiation. A tangent line is considered and the slope is calculated as the bearing chord stiffness, as described in the ASTM D5961 test standard. Following this stage, a nonlinear relation is developed as a result of the initiation and development of bearing damage in the laminates up to the ultimate bearing strength [33]. After its peak value, the stress rapidly decreases with an increase in the strain as a function of the excess propagation in the composite failure modes (matrix cracking, fiber breakage and delaminations), and the joint loses bearing capacity.

The data obtained from the bearing stress–strain diagrams are reduced to find the chord stiffness, offset bearing strength and the ultimate bearing strength for the whole test campaign. A comparison of the readings of the three configurations is summarized in Table 1. In general, the readings show good repeatability, with a coefficient of variation less than 10% for all the parameters under consideration.

It is well-established that decreasing the ply thickness delays the onset and propagation of interlaminar and intralaminar damage, such as delamination, matrix cracking, and fiber breakage, without the use of special matrices or changing the fiber microstructure [34]. The current comparison shows a limited reduction in the chord stiffness by introducing thin-plies inside the laminate. The reduction is 4% and 11% for both the alternative 1 and alternative 2, respectively. It is worth noting that the three laminated configurations were designed to have the same amount of fiber in the 0${}^{\circ }$, 90${}^{\circ }$, and $\pm 45^{\circ }$ directions, to ensure the same in-plane stiffness. However, the results show differences in the stiffness; moreover, this difference is dependent on the distribution of thin-plies inside the laminate. Similar results were reported by Moon et al. [24] for thin/thick plies under tensile loading.

**Figure 6:**
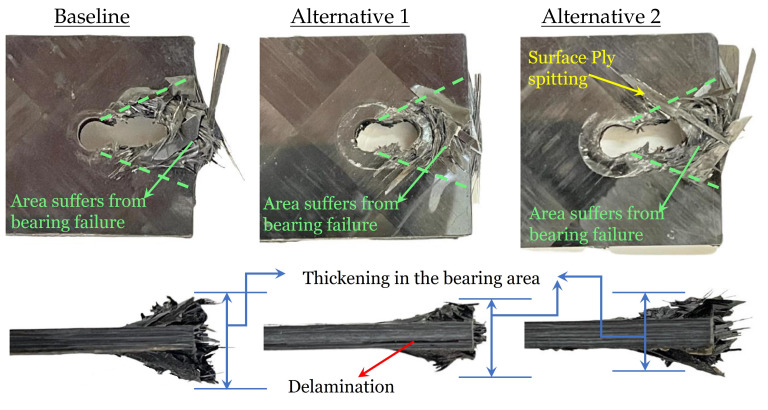
Examples of the failure mode associated by the three configurations.

On the other hand, the offset strength shows a slight improvement compared to the baseline laminate. The measured offset strength values of the alternatives (both 1 and 2) are approximately 5% higher than the baseline. However, the ultimate strength values show a significant improvement; 12% and 19% for alternative 1 and alternative 2, respectively. The improvement in the bearing strength (both offset and ultimate) can be justified by the in situ effect of strength as a function of ply thickness [35], the possible increase in the fracture toughness [36], the low stiffness measured in Table 1 that is expected to increase the failure strain at failure, and the tendency of earlier fiber breakage for thin-ply laminate [37]. All these facts can be used to characterize the improvements in structural response after using thin-plies in the laminate. However, it cannot justify the increased improvement of a certain alternative compared to another. In an attempt to understand the difference, the failure mode associated with each configuration is shown in Figure 6.

The images clearly show the bearing failure mode, with an obvious combination of all the well-known damage mechanisms (matrix cracking, delamination, and fiber breakage) in the area behind the bolt (the area associated with dashed green lines). There is no visual evidence of shear-out or net tension failure modes. The thickening of the laminate behind the bolt is governed by the in-plane and interlaminar shear, leading to the compression and buckling of materials as the load increases and the damage events accumulate [13]. This thickening is another evidence of the bearing mode. Delamination is only observed from the specimen edge in Alternative 1, where the a thick core of thin-ply laminate is designed in the middle of the specimen. The appeared delamination is usually close to the specimen edge, which agrees with the hypotheses that having a cluster of thin plies results in large delamination away from that cluster [31].

**Figure 7:**
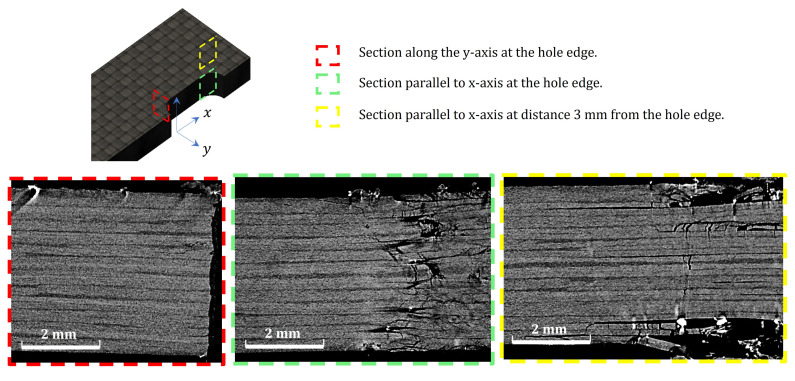
Computed Tomography scan of the baseline laminate.

**Figure 8:**
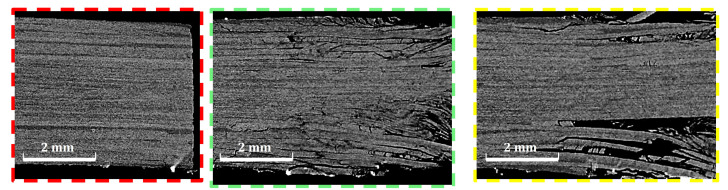
Computed Tomography scan of the first alternative (color code is in Figure 7).

**Figure 9:**
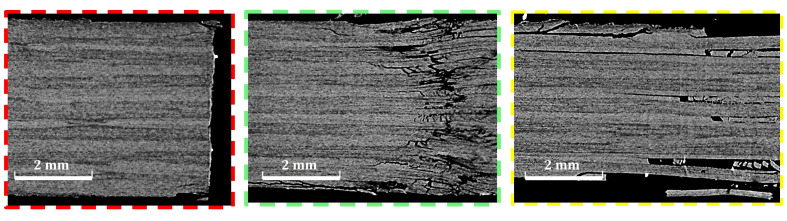
Computed Tomography scan of the second alternative (color code is in Figure 7).

To investigate the reason for the improved response in alternative 2 compared to alternative 1, a micro-computed tomography scan was performed for a specimen of each configuration after being tested. Figure 7, Figure 8 and Figure 9 show the results at three sections. The first section is marked by red dashed line. This section passes the hole center in the x–z plane. The comparison shows minimal delaminations at this plane, which confirmed the net-tension free damage mode, as was introduced in Figure 6. Even when this appears, it is extremely short, which indicates that edge delaminations occurred during the drilling process of the holes.

The second section (marked with green the figures) was designed to compare the delaminations and interlaminar damage at the end of the hole extension when the specimen lost most of its bearing abilities. The third section was designed 3 mm from section two to check whether the delaminations extend beyond the bearing area. For alternative 1, Figure 8, the core, made of pure thin plies, shows no delaminations. Instead, most of the energy absorbed by the specimen was consumed for larger delaminations at the surface layers made of thick plies. This observation justifies the delamination that appears at the specimen edge for alternative 1 in Figure 6. Obtaining this control over the damage propagation is the main responsibility of the improvement obtained in the ultimate bearing strength of 12%, as shown in Table 1.

On the other hand, for Alternative 2, as in Figure 9, the image at the end of the elongation zone of the bolt shows small delaminations at all the interfaces, leading to a loss in the bearing capacity of the specimen. At Section three, the more stable crack propagation shows that delaminations do not occur at every interface. Instead, they are distributed throughout the thickness, with a clear concentration at the matrix cracking developed through the $\pm 45^{\circ }$ thick layer. There is no evidence of matrix cracking inside the thin-plies, nor of delaminations in between two adjacent thin plies. This is driven by the higher fracture toughness compared to traditional ply thickness [36]. Again, this control of the damage sequence is assumed to be the main source of improvement in the ultimate bearing strength of 19%, as in Table 1. This result shows that improving the strength of each individual ply by, surrounded by two thin-plies (the in situ strength phenomenon), is of a higher advantage compared to designing a thick core of thin-plies at the specimen’s mid-plane for double-shear bolted-joint tests.

## 5. Conclusions

A set of experimental tests were conducted on CFRP composites to check the effect of inserting thin-plies inside the laminate configuration. The test specimens are either designed with fully traditional plies, with a core of thin-plies at the mid-plane, or with each normal ply being surrounded by two thin-plies to improve its in situ strength. The specimens were tested using a double-shear bolted-joints test and then monitored using a CT scan. The following conclusions can be drawn:

- All the specimens were confirmed to break under bearing mode with no effect of the insertion of thin-plies inside the laminates.
- The laminate designed with a core of thin-plies at the specimen mid-plane improved the ultimate bearing strength by up to 12% by controlling the damage inside the thick plies and leaving the thin-plies’ core undamaged.
- The laminate designed with the aim of improving the in situ strength of each layer by surrounding it by two thin-plies succeeded in improving the ultimate bearing strength by up to 19% as a result of controlling the damage inside the thick plies and leaving the thin-plies as damage-free areas inside the laminate.

## Figures and Tables

**Table 1:** Comparison of the bearing chord stiffness, offset strength and ultimate strength of the three configurations.

	Chord Stiffness		Offset Strength		Ultimate Strength	
	Mean (MPa)	CoV (%)	Mean (MPa)	CoV (%)	Mean (MPa)	CoV (%)
Baseline	1316	5.37	736	4.04	791	2.21
Alternative 1	1265	9.54	747	9.72	888	1.43
Alternative 2	1166	9.97	772	1.74	940	3.30

## Data Availability

Not applicable.
